# Nanoparticle delivery through the BBB in central nervous system tuberculosis

**DOI:** 10.1002/ibra.12087

**Published:** 2023-01-29

**Authors:** Anna Griego, Edoardo Scarpa, Valeria De Matteis, Loris Rizzello

**Affiliations:** ^1^ Department of Pharmaceutical Sciences University of Milan Milan Italy; ^2^ The National Institute of Molecular Genetics (INGM) Milan Italy; ^3^ Department of Mathematics and Physics “Ennio De Giorgi” University of Salento Lecce Italy

**Keywords:** central nervous system tuberculosis, infectious diseases, nanoparticles biodistribution

## Abstract

Recent advances in Nanotechnology have revolutionized the production of materials for biomedical applications. Nowadays, there is a plethora of nanomaterials with potential for use towards improvement of human health. On the other hand, very little is known about how these materials interact with biological systems, especially at the nanoscale level, mainly because of the lack of specific methods to probe these interactions. In this review, we will analytically describe the journey of nanoparticles (NPs) through the brain, starting from the very first moment upon injection. We will preliminarily provide a brief overlook of the physicochemical properties of NPs. Then, we will discuss how these NPs interact with the body compartments and biological barriers, before reaching the blood–brain barrier (BBB), the last gate guarding the brain. Particular attention will be paid to the interaction with the biomolecular, the bio‐mesoscopic, the (blood) cellular, and the tissue barriers, with a focus on the BBB. This will be framed in the context of brain infections, especially considering central nervous system tuberculosis (CNS‐TB), which is one of the most devastating forms of human mycobacterial infections. The final aim of this review is not a collection, nor a list, of current literature data, as it provides the readers with the analytical tools and guidelines for the design of effective and rational NPs for delivery in the infected brain.

## Graphical abstract

One of the hallmarks of human tuberculosis is the formation of granuloma (in blue)—a cluster of diverse immune cells formed in response to chronic infectious or noninfectious stimuli. While granuloma has been well characterized in its most physiologically relevant environment, namely, the lung, very little is known about the formation of granuloma within the brain. Interestingly, considering that the central nervous system (CNS) has high inaccessibility, coupled with a low immune surveillance, CNS might indeed represent a unique advantageous microenvironment where *Mycobacterium tuberculosis* can escape immune recognition, and thus evade host immunity. Nanoparticles targeting and bypassing the blood–brain barrier represents a unique chance of fighting brain tuberculosis, as standard antibiotics are less effective because of permeability problems.

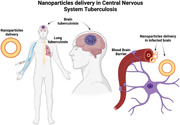

## 1 INTRODUCTION

The human body is a fascinating amalgam of several different and highly specialized compartments. Each of these has a specific function, strictly dictated by the cells composing it. Together with tissue specialization, we also evolved refined ways to overcome the issue of compartmentalization, especially in the form of signaling processes. For example, the cardiovascular system enables the transport of nutrients and gases even at the outermost periphery of tissues, and the endocrine system ensures communication between anatomically unconnected organs through the release of hormones. If we scale down by a few orders of magnitude in size, and thus describe the traffic inside a cell, we will come up against the same problem of compartmentalization, and strategies adopted to overwhelm it. This is probably one of the reasons why a cell should be considered an organism within another organism in terms of the complexity of behaviors. A revolutionary concept of cellular traffic control was put forth by the visionary Christian de Duve, and its cytonautic, a term used to exemplify the vesicles that are important for the intracellular transport of external uptaken material.^1^ Inside each cell (independently of which tissue it belongs to), there is a whole separated world where thousands of biochemical reactions and very dynamic trafficking take place.^2^ The problem of reaching a specific body and cellular isolated compartments, or an intracellular organelle, is a recurrent focus of all pharmacological studies. It is paramount for a drug to reach the desired molecular target without interacting with other molecules and macromolecules. In addition to pharmacologists, currently, material scientists are facing the same issue since the applications of materials in the biological and medical fields, the so‐called biomaterials, have significantly risen in refinement in the last decades of the twentieth century.^3^

The current scientific field includes several examples of biomaterials that are used daily in the clinic (and outside) including prostheses, coating for medical devices, contact lenses, wound dressings, sutures, cosmetic implants, nerve conduits, and vascular grafts.^4^, ^5^, ^6^ However, the advancement of knowledge in the life sciences, combined with the progression of new ways to manipulate matter, has yielded new and more sophisticated versions of biomaterials.

A significant stimulus for the design and development of previously unknown classes of biomaterials has specifically been provided by the advent of Nanotechnology, which enables fine control of the properties of materials at the nanoscale level.^7^, ^8^ Most probably, when in 1959 Richard P. Feynman, unanimously considered the father of Nanotechnology, declared that “there is plenty of room at the bottom,” he could never have imagined the revolutionary potential of his words, especially in the context of applying Nanotechnology to the development of biomaterials. By scaling down the design, the principles of biomaterials can be applied to engineer new ways of navigating the body. The aim will be to cross different biological barriers and target specific parts with the dual purpose of delivering therapeutic cargoes more efficiently and gathering functional information for diagnostic purposes.^9^ As a consequence, there are now biomedical nanomaterials for controlling tissue and cell growth, drug delivery systems, nanoscopic carriers, and various sensing devices. Moreover, biomaterials are applied to push biological control, at the single cell level, to recreate organ harvesting stem cell capability.^10^, ^11^

It is evident that a critical aspect in biomaterial design is the understanding of how the materials may control, and even tailor, biological systems, and how these outputs can be, in turn, translated into new material design.

Despite the growing literature data available in the field of NPs for biomedical applications, many issues still remain concerning their interaction with biological molecules in situ. This is mainly due to the complexity of the biological environment, together with a lack of translational information about the biological dynamics of nanomaterials. The scattered information describing the interaction between nanomaterials and biological systems hinders the chance to have established translational theories on the basic mechanisms of this phenomenon.

With all these premises in mind, the principal aim of this review article is to describe the long journey of NPs from the time of injection till reaching the BBB, one of the most controlled body‐environment, before accessing the brain parenchyma. To do this, we will describe all the possible interactions (both desired and random) occurring with human body components, starting from the molecular level, and up to the interaction occurring with cells and tissues, and finally the requirement for efficient BBB targeting, followed by access to the brain. Along with the biological journey and the multiple interactions, we will provide useful guidelines for the design of NPs targeting a desired outcome. The topic of NP–BBB delivery will be framed in the context of central nervous system tuberculosis (CNS‐TB), which is among the least common—yet the most devastating—forms of human mycobacterial infection. The mechanisms of BBB crossing mediated by *Mycobacterium tuberculosis* (*Mtb*), the etiological agent of human tuberculosis (TB), will also be discussed. We believe that this review will provide the readers a fresh perspective on an uncommon application of precise brain delivery for infection treatment on the one hand. On the other hand, the article will provide tools for understanding the analytical approach required to design active/effective NPs.

## 2 THE JOURNEY OF NPS WITHIN THE BODY

### 2.1 Preliminary considerations on NPs' toxicity

The biocompatibility of NPs is definitely a critical aspect in the context of healthcare applications. We are asked to produce something that interacts with the human body to exert a positive effect, with minimal collateral damage. The aim of this section is not to report the current knowledge of nanotoxicology, as many aspects of the mechanisms of NPs toxicity have been reported.^12^, ^13^, ^14^ We will make the preliminary assumption that each circulating NP must be biocompatible and clinically approved or made of generally recognized as safe (GRAS) material (i.e., its components are already being used and considered safe in different clinical contexts). It is thus not surprising why biomaterial scientists are reluctant to synthesize new materials, as scientists prefer to use already established clinically‐safe ones. Material scientists must consider opting for materials that have a short life in the biological milieu and that degrade into easy‐to‐metabolize components, especially in the context of intravenous injections (like polylactide, polyglycolide).^15^, ^16^, ^17^, ^18^ In this respect, it has been recently observed that the human body can perform even drastic degradation, digesting both “unbreakable” (e.g., carbon nanotubes) and biologically inert (PEO) materials.^19^, ^20^, ^21^

Nevertheless, we will focus on the issues concerning the “potential” toxicity of the NPs, discussing specifically which analytical approaches should be used, and further implemented, to address their safety. First, most of the tools used to investigate collateral damage are not universally appropriate. NPs' toxicity is usually assessed using cell cytotoxicity assays and, in most cases, on immortalized cell lines as this is a quick and easy way to determine critical toxicity. The standard cytotoxicity screens include (i) viability (by MTT), (ii) membrane damage (by LDH), (iii) oxidative stress (by dichlorofluorescein assay), and (iv) cellular metabolism (a measure of ATP levels) assays.^22^, ^23^ In this regard, it is worth noting that most of the cytotoxicity assays are based on the conversion of a given substrate by metabolically active cells, which is then molecularly transformed into a luminescent colorimetric readout. While providing useful information, all these assays fall short of determining whether the cells of interest are in cryostasis because of the treatment. Together with cytotoxicity, genotoxicity studies are also performed nowadays to determine any potentially hazardous effects on genetic materials.^24^, ^25^ This is usually based on comet or TUNEL assays. However, it is worth mentioning that all these methods do not provide a full picture of the potential harm in more complex systems. Alternative approaches have thus been proposed, such as tissue engineering models re‐creating some of the in vivo complexity.^25^, ^26^ Another way is to study cellular damage in depth by focusing on more refined investigations like autophagy, Nuclear factor kB (NF‐kB) translocation, endoplasmic reticulum (ER) stress, antiviral responses, or even adequate gene and protein screenings. Such methods can provide important information on several aspects, especially in the viewpoint to disclose unplanned outcomes (e.g., anti‐inflammatory activity, immune response, stress recovery, etc). A further improvement could be achieved by immunological evaluations to assess possible interactions with complement proteins, and the potential effects on immune cells, trying at the same time to embrace the more established theories of immunology (e.g. the danger model).^27^, ^28^ Even though in vitro methods provide a basic characterization of NPs, discrepancies between in vitro and in vivo studies are too often reported, demonstrating not only that traditional in vitro assays remain limited but also that in vivo experiments represent the prudent choice for exploring NPs' toxicity more comprehensively.

**Figure 1:**
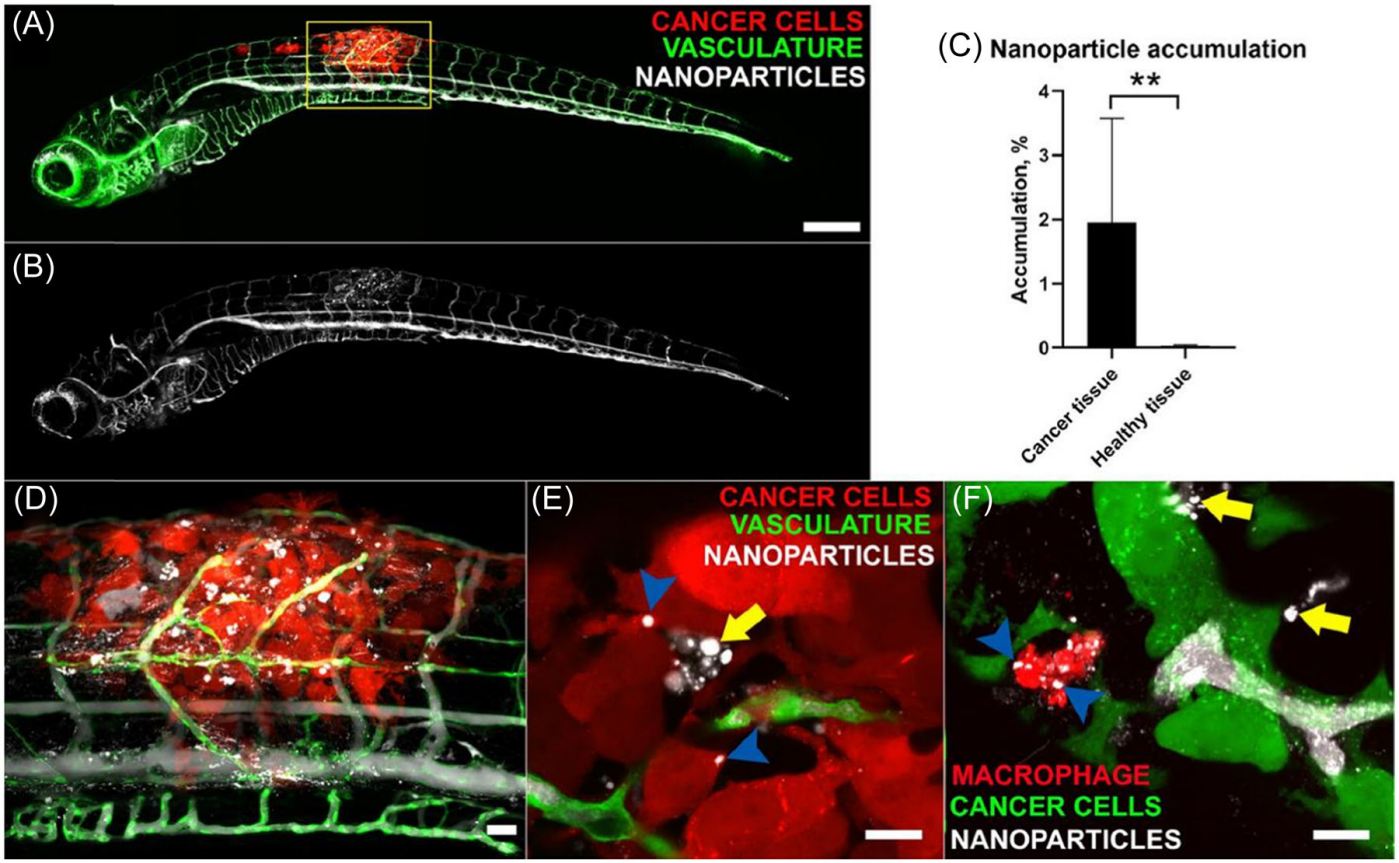
Accumulation of PEG‐PDPA NP in the area of the tumor. (A) Nanoparticles (white) injected intravenously can be seen flowing in vessels in the zebrafish (green) and selectively accumulating in the tumor area (red). The inset shows the image in the transmission channel. The same image is shown in (B) only in the NP channel to highlight the local accumulation. A quantification of NP accumulation based on fluorescence is shown in  (C). (D) High magnification of the tumor area (yellow box in A). (E) Confocal slices in which the injected NPs (white) appear to be inside cancer cells (red, blue arrowheads), while others are free in the intercellular spaces (yellow arrows). (F) Confocal stack showing a macrophage (red) near B16 cancer cells (green) that took up NPs (white, blue arrowheads). Other NPs are free outside macrophages and in the vicinity of cancer cells (yellow arrows). Scale Bars: (A, B) 200 µm, (D) 50 µm, (E, F) 10 µm. NP, nanoparticle. Reprinted from  Kocere et al.^34^ Copyright (2020), with permission from Elsevier. [Color figure can be viewed at wileyonlinelibrary.com]

Again, new developments are available, as we now have access to new animal models that facilitate high‐throughput (HTP) screenings. Some examples are flatworms, slugs, and zebrafish.^29^, ^30^, ^31^ In particular, the latter is emerging as a new non‐rodent vertebrate model for assessing the use of NPs in cancer or infection treatments (Figure 1).^32^, ^33^ Zebrafish models are easy to handle and relatively cheap to maintain. They also allow for quite sophisticated whole‐body and high‐resolution imaging with faster and more efficient outputs.^34^, ^35^ However, they are still physiologically simple and fall short in recapitulating the biological complexity of the human body.

### 2.2 NPs interfacing with biological barriers: an overview of the intra‐body itineraries

The human body is a highly compartmentalized system with several components creating very different local compositions. Yet, there are several highly gated transport mechanisms that regulate signaling and metabolism across the different organs. These barriers inevitably hinder nanomaterials entry into and diffusion within the body, and consequently, new strategies are required to establish new design principles for nanomaterials. Biological barriers cannot really be grouped into classes and each specific class is eventually rather difficult to describe because most of them share common pathways. However, it can be roughly categorized that there is a fine control over the presence of external molecules at molecular, cellular, tissue, and organ levels. This mostly depends on the specific method of administration, so that one barrier may act before another one, while some of them may be completely overcome.

#### 2.2.1 Interactions with blood components

**Figure 2:**
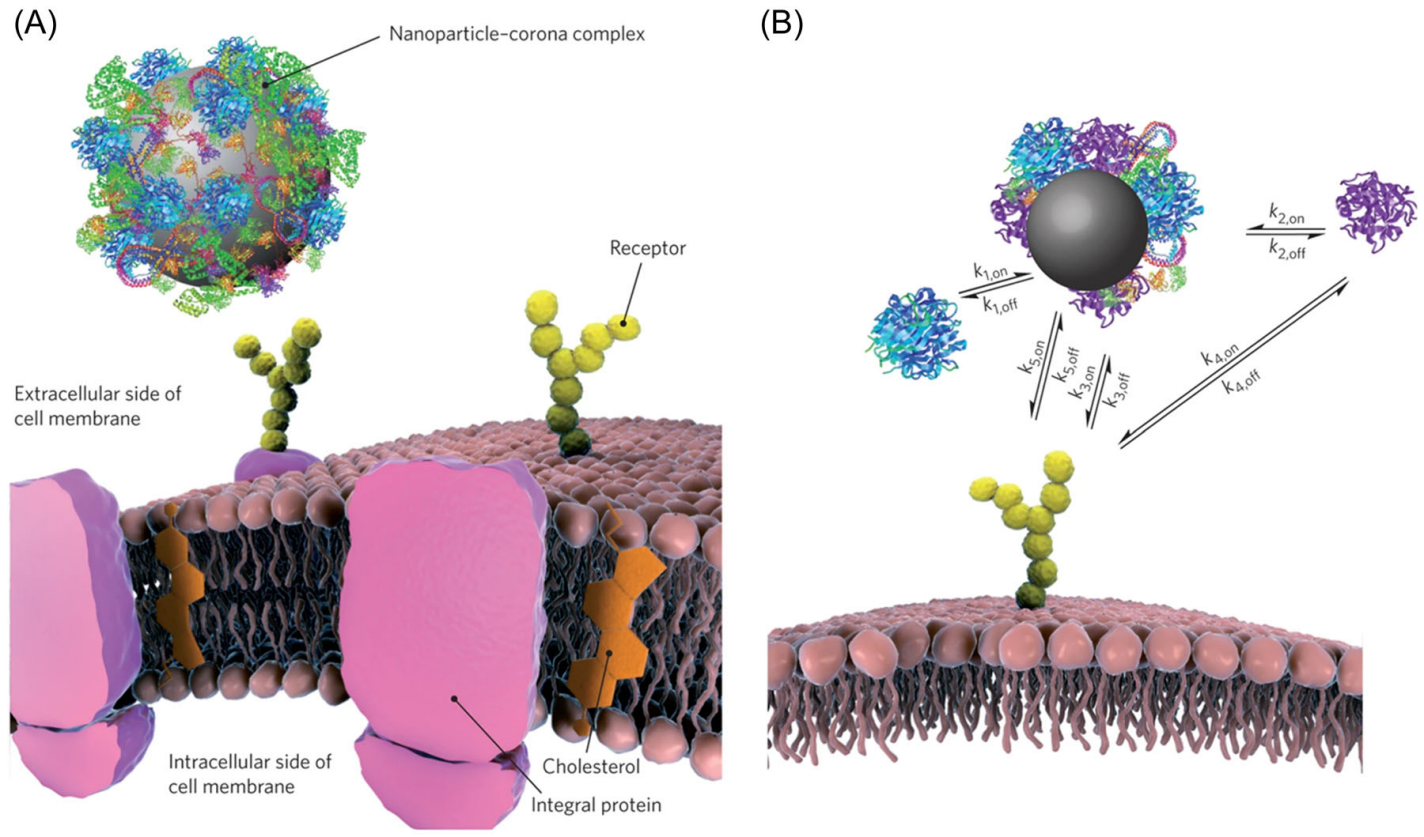
(A) The nanoparticle–corona complex interacting with a membrane receptor. (B) Relevant processes (arrows), in both directions (on/off), for a nanoparticle interacting with a receptor. Biomolecules in the environment adsorb strongly to the bare nanoparticle surface (k_1_), forming a tightly bound layer of biomolecules, the “hard” corona, in immediate contact with the nanoparticle. Other biomolecules, the “soft” corona, have a residual affinity to the nanoparticle–hard corona complex (primarily to the hard corona itself), but this is much lower, so those molecules show rapid exchange with the environment (k_2_). If sufficiently long‐lived in the corona, a biomolecule may lead to recognition of the nanoparticle–corona complex as a whole by a cell membrane receptor (k_3_). The same biomolecule can also be recognized by the receptor (k_4_). If present, the bare surface of the nanoparticle may also interact with cell surface receptors (k_5_) or other constituents of the cell membrane. Reprinted by permission from Springer Nature: Monopoli et al.^45^ Copyright 2012. [Color figure can be viewed at wileyonlinelibrary.com]

For the journey to begin, NPs must be effectively carried to their specific target. Nevertheless, an important fact that should be kept in mind is that the journey will be significantly affected by the method of introduction within the body, as the NPs will interface with different barriers. The administration routes include subcutaneous, sublingual, and topical inoculations (e.g., ocular, cutaneous, and transdermal), with intravenous (IV), intramuscular, and oral administration routes being the most common ones. For the sake of simplicity (as it would be extremely cumbersome to consider all the possible ways of administration), we will focus only on the IV injection route. Here, the very first molecule that NPs come into contact with inevitably is water. This fact should not be overlooked. Water is the second most common molecule in the universe, second only to molecular hydrogen, and it is surely the most important in component of life. It is well known that water molecules interact with each other *via* hydrogen bonds, forming an average of 3–3.5 bonds per molecule in the liquid state.^36^ Any guest molecule in water will induce perturbations to the water network, and they will attract or repel each other depending on how the guest interacts or does not interact with water molecules. It goes without saying that hypothetical hydrophobic NPs will not be able to enter the water network and they will be repelled by it (hence be attracted to each other) with forces whose magnitude in some cases exceeds the attraction in the vacuum.^37^ Indeed, it is not surprising why most NPs are either soluble or have a hydrophilic surface. Another important aspect is that the interaction between the NPs and water has extreme consequences in terms of interaction with other molecules, as this also occurs *via* hydrogen‐bonding perturbation. The molecular surface nature of the NPs will thus govern this interaction in a specific way. Traditionally, this approach has been adapted to the existing repertoire of theoretical models and experimental approaches created by surface scientists. However, these are often based on the simplified notion that surfaces are two‐dimensional entities, atomically ordered, while hydrogen bonding has defined and directional bond lengths and angles.^36^ This means that, in addition to the chemical nature, supramolecular forces can also be controlled by the three‐dimensional conformation of the NPs. Within any biological fluids, and particularly in blood, the fluid phase also has several other solutes including salts, small metabolites, and proteins. Without going into the physicochemical details on how such complex solutions work, one important aspect to consider is that biological liquids are almost saturated in salts. This means that any electrostatic interaction is inevitably screened by the large amounts of electrolytes.^38^ One level up in molecular size, and the next encounter, results in dealing with proteins. The “fouling” problem (i.e., the inevitable interaction between exogenous materials and proteins) has dominated Biomaterial Science for many decades, due to its critical impact on the final delivery of the NPs. First described as protein “adsorption” in the 1950s and the 1960s,^39^, ^40^ the concept of protein corona has rapidly evolved over the recent years, with the studies of Kenneth Dawson (Figure 2).^41^, ^42^ At the core of this biological phenomenon, there are three key concepts. First, all NPs will interact with plasma proteins such as albumin (HSA), apolipoproteins, and immunoglobulins.^43^, ^44^ Second, these interactions will result in the formation of layers of proteins (to different extents as a function of the specific NPs physicochemical properties), which are classically defined as “hard corona” and “soft corona”, whereby the first comprises an inner protein layer that is strongly bound onto the NPs surface, while in the second, there is a dynamic exchange of proteins between the NPs surface and the environment.^45^, ^46^ There is evidence that proteins characterizing the “soft corona” are covalently bound to the “hard corona” rather than the surface of the NPs.^47^ It is also worth underlining that the majority of the studies focused on ex vivo characterizations of the protein corona. However, it is now clear that although the final amounts of protein present on the surface of the NPs in vivo and ex vivo are correlated, their relative abundance and variety are different. The reason for such differences lies in the absence of blood flow dynamics or interactions with cellular components outside of the body.^44^, ^48^ Third, the NPs covered by proteins will behave as a completely different nano‐object because they will possess new physicochemical properties.^45^ This latter event has also been found to be the main cause of the dissimilar toxicity of the same batch of AuNPs incubated in two different cell culture media.^49^ Concerning the nature of NP–protein interactions, noble‐metal hard NPs (e.g., gold and silver) strongly interact with cysteine‐rich peptides/proteins in a quasi‐covalent manner. This is because the thiol moiety is a soft ligand, with the highest occupied molecular orbitals of high energy, and will strongly bind soft cations with low unoccupied molecular orbitals of low energy. On the other hand, the binding between soft NPs and proteins is relatively weak, as it is mainly a result of Wan der Waals interactions. However, this binding is highly dynamic, and it varies in conditions of health and disease. Recent work has demonstrated that the protein corona formed around NPs administered in humans can be used as an analytic tool to investigate the circulating proteomes.^50^

Because of the fouling process, many efforts have been focused on finding strategies to avoid undesired or uncontrolled/unspecific protein adsorption. In this context, a typical approach is the use of a specific coating that comprises the appropriate physical and chemical features to interact with water more strongly than with proteins. One of the best solutions is the use of poly(ethylene oxide) (PEO), commonly known as poly(ethylene glycol) (PEG). The antifouling property of PEO is due to its chemical nature. PEO has the correct electron–acceptor characteristic to promote water association and to create an energy barrier that prevents protein (or any other soluble polymer) interaction.^51^, ^52^ The non‐fouling characteristic can be increased by confining PEO chains within dense brushes that create a steric repulsion that prevents the protein from approaching the coated NPs. This behavior has been observed in several other polymers and surface coatings like phosphorylcholine,^53^ hydrophilic polysaccharides,^54^ and poly‐amino acids.^55^ However, the PEO‐based antifouling approach is also inevitably reductionist, so that complete biological inertness of the NPs is impossible, albeit significantly reduced. Clearly, the flip side is that the presence of proteins on the surface of the NPs could also prevent nonspecific cellular uptake,^56^ and could be used to increase NPs targeting or reduce cytotoxicity.^57^

One of the undesired effects of NPs covered by plasma proteins could be a systemic and uncontrolled immune response. In the immunological field, the fouling problem is known as opsonization. Immune‐regulating proteins can opsonize (i.e., cover) the NPs’ surface with more (or less) affinity. Among these, there are immunoglobulins and complement proteins (named after the original observation that some proteins “complement” with antibodies in the bacteria lysis). Due to the high binding specificity of immunoglobulin, it is quite unlikely that NPs may be opsonized. On the other hand, complement‐mediated recognition may occur. In particular, the complement consists of a pool of more than 30 serum proteins that are in the form of inactive precursors (i.e., the zymogen) within the bloodstream. In this framework, the circulating NPs are likely to interact with the complement protein C3 because it has an affinity to several non‐self materials, with consequent possible activation of the complement system. In particular, there is evidence that charged sulfide, lipids, and cyclodextrine‐modified NPs are more likely to activate the complement system compared to their respective unmodified counterparts,^47^, ^58^ while PEG coatings were found to reduce the complement activation.^59^ An NP‐induced constitutive activation of C3 may indeed lead to mild and transient reactions, also known as C activation‐related pseudo‐allergy (CARPA), as reported for Doxil.^60^, ^61^

**Figure 3:**
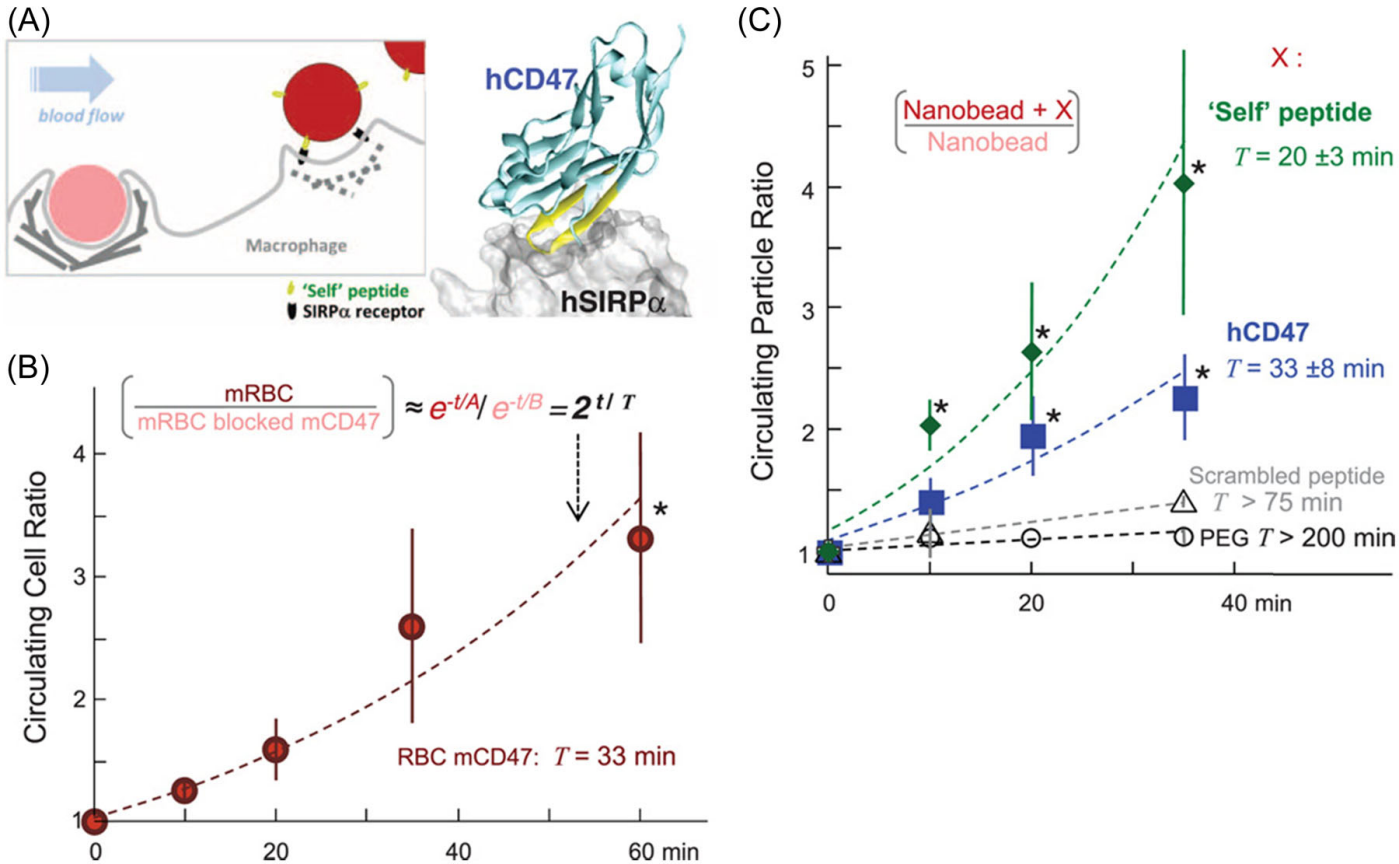
Self‐peptide and hCD47 prolong the circulation of nanobeads in NSG mice. (A) Competitive circulation in which two colors of nanobeads or cells injected into the same mouse are flowing with blood and being cleared by a splenic macrophage (left) or else recognized as self and released (right). (B) Competitive circulation experiment in which mRBCs from NSG mice were either blocked with anti‐CD47 or not and were also opsonized with excess mRBC‐specific antibody before cells were mixed together and injected into the tail vein. (C) Circulation experiments used 160‐nm polystyrene beads with covalently attached streptavidin incubated with biotinylated versions of one of the following: synthetic self‐peptide; recombinant hCD47; or negative controls of either Scrambled peptide or PEG. From Rodriguez et al.^63^ Reprinted with permission from AAAS. [Color figure can be viewed at wileyonlinelibrary.com]

The protein fouling of NPs can thus be associated with an immune response, and the human body differentiates endogenous from exogenous material through specific immune‐modulators motifs. Discher and colleagues have exemplified this approach by using NPs showing the self‐peptide extracted from the CD47 receptor (Figure 3). They observed that this “don't eat me” signal on the particles prevents inflammation, inhibits microparticle phagocytosis, and promotes persistent circulation of virus‐like NPs in vivo.^62^, ^63^ Such an immune modulator effect extends beyond the molecular level, and it is an exemplary approach to how molecular‐level interactions control more complex biological processes.

These are examples of how synthetic strategies may be used to modulate a desired biological outcome that is, in this case, immune regulation.

**Figure 4:**
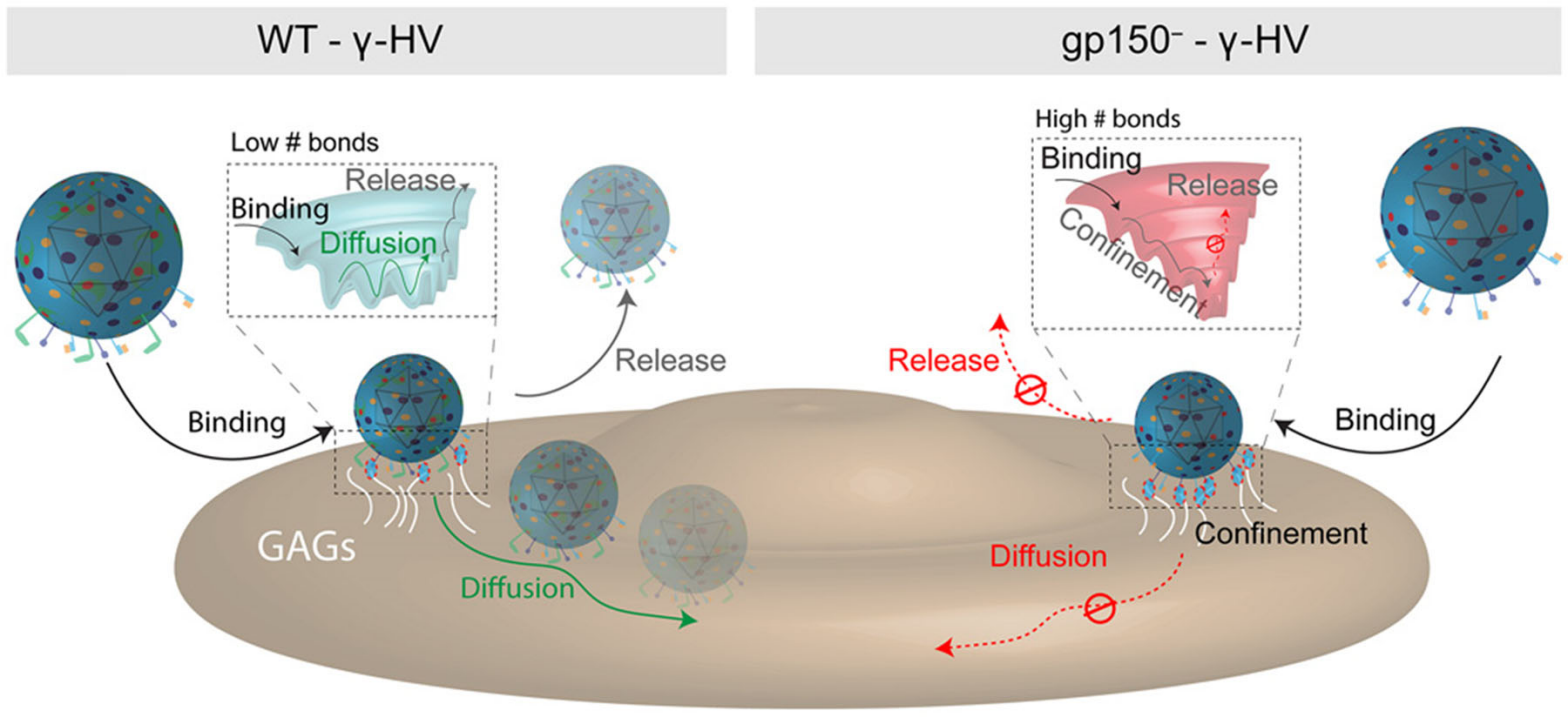
Free‐energy landscape describing the first binding steps of MuHV‐4 and gp150− to cell surfaces. (Left) After landing on the cell surface, the HV particle binds to GAGs. gp150 acts as a binding regulator by maintaining the number of foothold low, enabling the virus to diffuse laterally to seek specific receptors. (Right) gp150− virion lacking this regulatory element increases its adhesion to cellular surfaces, preventing the virus from undergoing lateral diffusion. The multiple bonds, preventing the virus from undergoing lateral diffusion and release, kinetically and thermodynamically stabilize the virus. Courtesy of M. Delguste et al.^64^ [Color figure can be viewed at wileyonlinelibrary.com]

The design of NPs is crucial in the context of overcoming the molecular barriers of the interactions occurring at the solid–liquid interfaces. The water molecules as well as the huge number of proteins present in the bloodstream will significantly impact both the NPs stability in situ, as well as their final fate as a function of uncontrolled opsonization. The final targeting efficiency may thus be strongly affected, resulting in an overall failure in correct delivery and effectiveness (Figure 4).

#### 2.2.2 Cell targeting and NPs uptake

**Figure 5:**
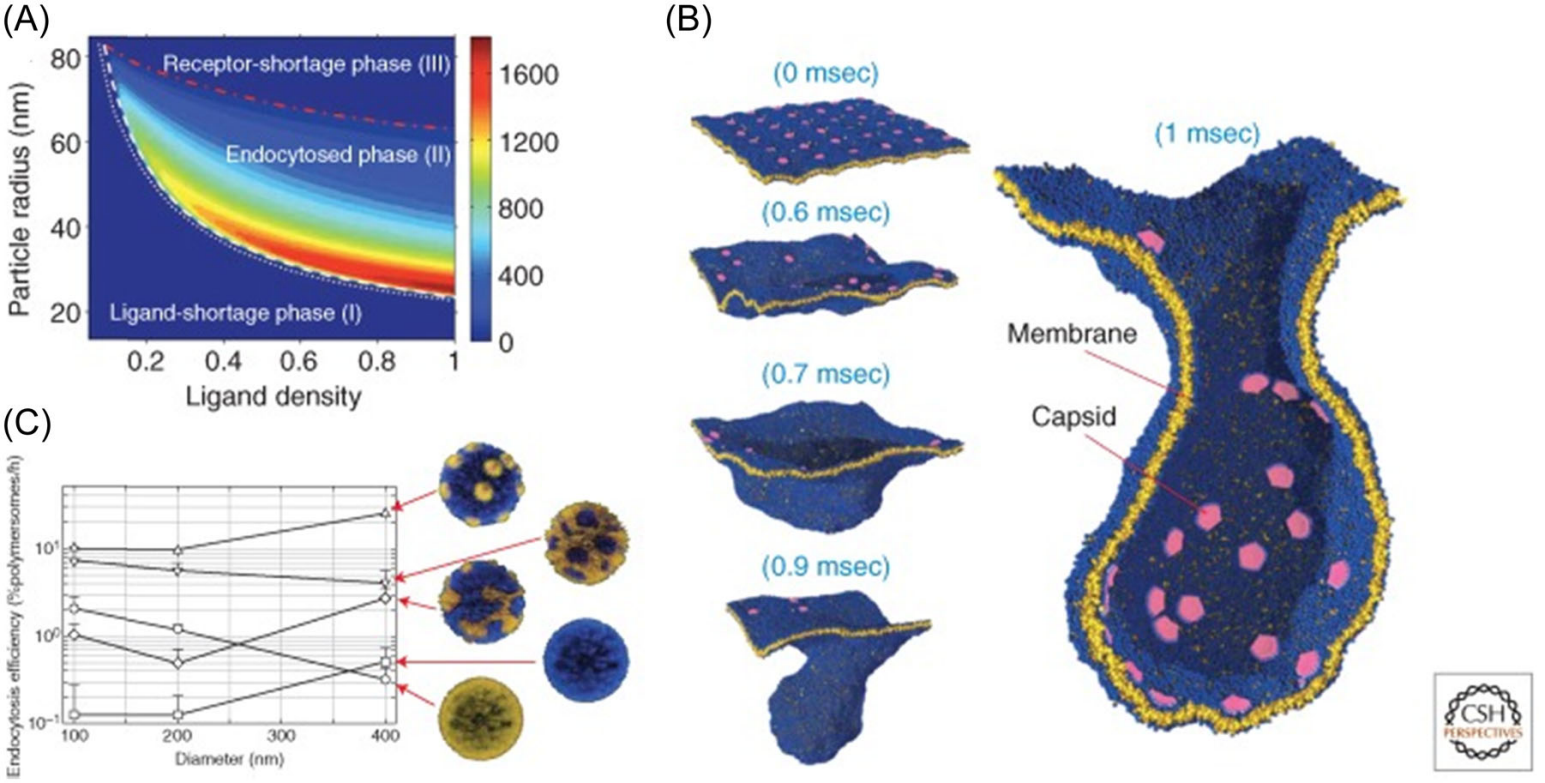
(A) A two‐dimensional (2D) phase diagram of the nanoparticle radius ligand density plane characterizes the interrelated effects of particle size and ligand density on the cellular uptake. (B) Coarse‐grained simulations of curvature‐inducing proteins bound on membranes at different times show that a membrane‐bound protein cluster drives the formation of vesicles, whose size is controlled by the local curvature uptake. (C) Endocytosis efficiency as a function of the polymersome diameter for different patchy cell‐active (gold) and cell‐inert (blue) nanoparticles. Figure from  Akinc and  Battaglia.^74^ [Color figure can be viewed at wileyonlinelibrary.com]

According to the previous descriptions, circulating NPs will interact with water and plasma proteins upon IV injection. However, they will also come into contact with both circulating and tissue cells, with a possible internalization event. In this context, it might be useful to mention that all the interactions that NPs have at this level certainly affect their capacity to bind the target of interest. This is a crucial topic in the current biomedical research because good targeting needs to be specific for the site of interest and this is necessary to avoid undesired adverse effects and prevent impairment of the fate of NPs when circulating within the human body. As introduced back in time by Paul Ehlrich, this specificity is related to the ability of tailor‐made macromolecules eventually present onto NPs surface, known as ligands, to bind selectively receptors expressed on the cells of interest. This concept has mainly been used and a high number of targeted strategies have been developed mainly based on the monovalent interaction between ligands and receptors.^65^, ^66^ In this unique recognition process, however, there are few chances for this interaction to be completely selective, especially for ligands with high affinity for their receptors. This is mainly because most of the time receptors are not solely expressed on the cells of interest, but they are present at low densities in other tissues. In this framework, the necessity to use receptor densities as a discriminating factor for cancer therapies has led to the use of multivalency as a crucial solution to address the selectivity requirements.^67^ Multivalency is based on the simultaneous interactions of multiple ligands with multiple receptors giving rise to peculiar behaviors not observable in the corresponding monovalent binding.^68^ This mechanism is very well known in nature as several aspects of cell biology are regulated by multivalency, such as viruses and bacterial infections (Figure 5),^64^, ^69^ DNA modifications, or antibody‐mediated processes.^70^, ^71^ Mammen et al.^72^ recognized the importance of this principle in the design of constructs able to quickly respond to receptors concentrations. However, a uniform and comprehensive theoretical explanation for the correlation of multivalency with selectivity was first proposed by Martinez‐Veracoechea and Frenkel some decades later.^73^ Here, the authors explain that having multiple binding arrangements between ligands and receptors is a *conditio sine qua non* to achieve selectivity as a function of the number of receptors. However, an on‐off regime can be exclusively established when the single ligand–receptor bond is sufficiently weak.

On the basis of the above information of ligand–receptor interactions, we should move on to analysis of which cells the circulating NPs will interact with. The first level of interaction is likely to occur with the most abundant cells in the bloodstream, namely, the red blood cells (RBCs). Not much literature data are available on the NP–RBC interaction. Complex theoretical simulations, where the cell hydrodynamics was modeled with particles' Brownian motions in microcirculation conditions, confirmed the crucial role of RBCs in NPs dynamics.^75^ Interestingly, the motion of RBCs seems to enhance the dispersion of NPs, which were predicted to preferentially group at the edge of the vessel, under flowing conditions. This phenomenon can be explained by a combination of hydrodynamic interactions with a volumetric exclusion effect of the RBCs.^75^

This is an important aspect since RBCs will affect the journey of the circulating NPs, which will probably avoid the lumen of vessels, and preferentially come into contact with the endothelium, thus possibly interacting with endothelial cells more efficiently. In this scenario, model human umbilical vein endothelial cells (HUVECs) were found to engulf different polystyrene NPs (in the size range of 100 nm).^76^, ^77^ Interestingly, the NP‐cell association was related to the quantity of protein adsorbed onto the NPs’ surface, rather than the identity (i.e., the type) of a specific protein present. In particular, NPs with a bigger protein corona were uptaken more efficiently by HUVEC cells, compared to the same NPs with a small (or completely lacking) protein layer around them.^77^ This further confirmed how protein corona changes the characteristics, and indeed the final fate, of nano‐sized circulating NPs.

During their “trip” through the systemic circulation, the NPs are likely to interact with the reticuloendothelial system (RES), also known as the mononuclear phagocyte system (MPS).^78^, ^79^ This is a significant pool of macrophages present in the spleen, liver, bone marrow, lungs, and lymph nodes. Its main role is to provide defense against possible invasion of microorganisms, toxins, and viruses.^80^ Macrophages may uptake the circulating NPs, especially those opsonized by complement proteins, thus increasing the rates of clearance and decreasing their biodistribution/bioavailability. Similarly to the NP–protein interactions, also, in this case, specific strategies have been developed to avoid RES clearance. As expected, PEG functionalization represented the best choice.^81^ However, we are still far from uncovering a general strategy for bypassing the RES clearance. There is evidence that more than 50% of the injected NPs ended in the liver and spleen after 48 h, even though they were highly PEGylated. The shape of the NPs is an important parameter for RES uptake.^82^ In particular, NPs are successfully internalized when they have a spherical shape (with a normalized curvature Ω ≤ 45°), while the speed of phagocytosis is inversely correlated to Ω. Ellipsoidal NPs with Ω > 45°, on the other hand, are not internalized. Moreover, NPs with a worm‐like structure are internalized much slower by alveolar rat macrophages, compared to spherical NPs. This may be explained by the high dominance of low‐curvature (i.e., flat) regions in the “worms” (Ω = 87.5°) over the only two high‐curvature regions (Ω = 2.5°), represented by the extremities of the structure.^83^

These examples highlight the complexity of the phenomena involved in the interactions occurring between NPs and the physiological components of the circulating system. It is also evident that good NPs should have a spherical shape, and an overall neutral charge, to avoid uncontrolled aggregation and/or nonspecific cellular internalization.

The mechanisms of NPs' uptake by cells, and the specific molecular pathways involved, are crucial topics of discussion, especially from the viewpoint of the final sorting. This is a highly debated and quite complex issue, where biology merges with physics, biochemistry, and molecular biology. The main question is how the NPs interact with the plasma membranes and what is the preferential uptake process.

The most well‐known mechanisms of NPs entry into the cell are phagocytosis and/or endocytosis. The former is performed by specialized/professional phagocytes, namely, mast cells, macrophages, neutrophils, dendritic cells, and monocytes. The process starts with an event of recognition between an extracellular ligand (usually functionalized on the surface of the NPs) and specific plasma membrane receptors present in the immune cells. Therefore, a signal cascade event leads to the local recruitment of actin filaments that, in turn, reassemble and distort the plasma membrane, with the resultant formation of membrane protrusions engulfing the NPs. These plasma membrane extensions will result in the formation of phagosomes, with the final dimensions dictated by the specific size and shape of the NPs (Figure 5). The phagocytosis of NPs may be driven by the opsonization of complement proteins (as described previously). These proteins will be, in turn, recognized by their respective complement receptors on the plasma membrane of immune cells. Several reports have already demonstrated that NPs may be engulfed in phagosomal vesicles with a final size of c.a. 1 μm.^84^, ^85^

Unlike phagocytosis, endocytosis is not delimited to professional cells, as it is ubiquitous in almost all eukaryotes. Also, endocytosis requires specific binding between an external molecule and its respective plasma membrane receptor. Then, the engulfment process may follow different pathways, depending on the main involved proteins, such as Clathrin, Caveolae, RhoA, CDC42, ARF6, and Flotillin.^74^, ^86^

In addition to phagocytosis and endocytosis, macropinocytosis could be another possible mechanism by which NPs enter cells. It involves the uptake of huge amounts of external fluids thanks to the extrusion of sheet‐like lamellipodia from the plasma membrane. The consequently formed vesicle, named macropinosome, has a size in the range of 1–4 μm. While both phagocytosis and endocytosis start with ligand–receptor binding, macropinocytosis is independent of this event.

It is worth pointing out that the physicochemical characteristics of the NPs play a pivotal role in the discrimination of the specific uptake process involved. Concerning the size, there is considerable evidence showing that smaller NPs may access the cells more efficiently compared to bigger NPs.^87^, ^88^, ^89^ However, this scenario looks slightly difficult to categorize, mainly because of the different approaches used to analyze and quantify the collected data. In this context, a simple normalization of the fluorescence signal of different‐sized SiO_2_ NPs would suggest that the role of size is negligible in the uptake,^90^ which has been confirmed by similar studies.^91^, ^92^ In this case, a combination of flow cytometry and UV–visible spectroscopy assays confirmed that the size of poly(2‐methacryloyloxyethyl phosphorylcholine) (PMPC) and poly(ethylene oxide) PEO polymeric vesicles (known as polymersomes) is not a fundamental parameter for endocytosis in human dermal fibroblast (HDF) cells.^93^ On the other hand, the efficacy and strength of the binding between the HDF and the NPs seem to be much more fundamental in the uptake efficiency, rather than size itself.

In addition to size, the shape/geometry of the NPs is another parameter that can be easily tuned during the synthesis of nanomaterials. While the observations on the NPs size are not in agreement, the literature data available on the effects of shape on cellular uptake are rather consistent. In particular, spherical NPs (AuNPs) seem to be engulfed much faster than tubular structures (Au nanotubes), independent of the size and/or the external functionalization of tubes.^94^ This has been very recently confirmed also by Robertson et al.,^95^ who explored the degree of uptake of polymeric vesicles and tubes. By using flow cytometry assays, the authors discovered that the internalization of rhodamine‐labeled tubular polymersomes yields a profile with two‐phase kinetics: immediately after the incubation, the tubes bind to the surface of the cells (neutrophils and FaDu cells were tested) without being engulfed; in the second step, they are slowly internalized. On the other hand, spherical polymersomes were rapidly internalized by the same cells.^95^ Among the different‐sized tubular structures, carbon nanotubes with a specific size of 320 × 26 nm (length ×  radius) were found to be taken up more efficiently compared to rods of other sizes.^96^ It is interesting to note that most of these tubular structures enter the cells via endocytosis‐independent pathways, namely, through physical interactions, thus acting like “needles” puncturing the plasma membrane. From the previous examples, it is evident that the shape of a given NPs may strongly control the way they interact with cells and their consequent internalization.^82^ This discovery is leading to the design of more effective drug delivery systems able to evade, for instance, immune system activation.^83^

In addition to size and shape, another important parameter is the surface topology of the NPs, which refers to how molecules and macromolecules are arranged on a given interface, forming specific patterns and domains. In this respect, interface‐confined separation of copolymers has recently been used to design materials with controlled surface topology. For instance, the specific mixing of cell‐inert with cell‐active polymers may lead to the formation of controlled patterns. These have a size range from tens to hundreds of nanometers, either on NPs or on microporous scaffolds, which drastically affects cellular processes. Patchy NPs were observed to enter cells more effectively than their pristine counterparts.^95^, ^96^ Moreover, the surface topology may affect protein adsorption and, consequently, the cellular response.^97^ It is thus evident that precise targeting is not only a molecular affair, as it can be significantly implemented using supramolecular engineering to create structures that combine molecular recognition with mesoscopic ones.

### 2.3 The tissue barriers

The assembly of cells into tissues involves a highly orchestrated set of biological events. Cells must be exposed to the appropriate mechanical and chemical stimuli and the correct nutrient supply. They must be able to communicate among themselves and rearrange themselves according to the function they must perform. The ability of the NPs to go across such a complex system can be addressed by studying all the characteristics implied by its diffusion. The extravasation from the blood capillaries of the NPs is followed by interaction with the highly viscous matrix present in the extracellular space (ECS). The ECS, or interstitium, is commonly composed of a network of collagen fibers and other molecules such as hyaluronic acid and nonstructural proteins such as proteoglycans. Altogether, this is called the extracellular matrix (ECM), which is characterized by a fluid phase consisting of water, electrolytes, nutrients, and some plasma proteins. The NPs have to pass through this complex and dynamic environment. The particles will move by Brownian random motion in between the spaces of the network structures, and it has been estimated that the fluid flows from the capillary at a low velocity of 0.1–4 m/s.^98^ Thus, the movement is influenced by the matrix components^99^ because of steric (collision with matrix fibers), hydrodynamic (low diffusion of water molecules due to proximity with fibers), and electrostatic interactions (for charged particles).^98^, ^99^ The interactions with the different configurations, as well as the physicochemical properties of the ECM components, will determine the direction, speed, and distance of NPs transport with a specific size and/or charge. If we take a closer look at these interactions, the electrostatic and hydrodynamic interactions are probably the most important ones.

Concerning the role played by electrostatic and hydrodynamic interactions, the diffusion in a fibrous media was the first model where the random walk of NPs was studied. In particular, it was observed that when the particle size is significantly smaller than the diameter of the fiber, the hydrodynamic interactions are negligible.^100^ However, when the particle size is comparable to or even larger than the fiber diameter, hydrodynamic hindrance slows down the mobility of the particle more than two‐fold, and hydrodynamic interactions become crucial. Furthermore, it has been demonstrated that neutral particles might diffuse faster than cationic ones.^98^, ^101^ Thus, for large fibers, electrostatic repulsion might not be significant and, thus, charged particles (having charges similar to that of the fibers) will have the same diffusivity as neutral particles.

At the same time, the size of the NPs can limit diffusion within the ECM and tissues. Nonetheless, enhanced production of growth factors, signaling, and adhesion molecules at the tissue level can also further change NPs penetration within the tissue. Steric interaction can be considered negligible for small molecules, whereas it can hinder large NPs movements and thus only allows for their local drug release.^102^ Uniform diffusion of small NPs (64 nm in diameter) has been observed to occur through neocortical ECS channels. In this case, larger NPs transport may occur by different mechanisms, like entropic barrier transport or reptation. Size and physicochemical properties play an important role in the overall accumulation and penetration depth into tissues. For instance, polymeric (i.e., soft) NPs can penetrate the hard lung granuloma (a hallmark of TB in humans), which is one of the hardest‐to‐reach body compartments for many drugs.^103^

NPs larger than 100 nm are trapped in the ECM between cells because they are not able to extravasate far beyond the blood vessel. On the other hand, smaller NPs (20 nm) are able to deeply penetrate within the tissues.^104^

As expected, NPs shape also plays a major role in tissue distribution: solubilization, circulation time, and cellular uptake are parameters that all depend on the shape of the NPs. In particular, rod‐shaped or filamentous materials have been shown to better penetrate tissues compared to their spherical counterparts. Additionally, the circulation lifetime of rod‐shaped micelles is 10 times longer than that of spherical micelles.

The surface chemistry of the NPs also dictates their behavior in the surrounding environment. Hydrophilicity, surface charge, immunogenicity, in vivo circulation, bio‐distribution, and intracellular bioavailability are parameters that can all be modified by changing the arrangements of the chemical groups on the surface of the NPs.^105^ The ability to modify the NPs surface can be an effective way to control the interface between NPs and the biological systems that they interact with. This can lead to the development of NPs able to maximize therapeutic efficacy while minimizing unfavorable side effects. In this framework, Battaglia and co‐workers showed that the modification of the external surface of polymersomes (obtained by changing the ratio of the two main polymers) significantly influences cellular uptake through a super‐selectivity process.^91^, ^93^, ^104^

### 2.4 *Mycobacterium tuberculosis* and CNS‐TB

*Mycobacterium tuberculosis* (*Mtb*) infection commences when air droplets (1–5 μm size), containing a few tubercular bacilli (estimated at 1–10), are inhaled by healthy individuals. Although most of the inhaled bacilli will be cleared by the innate immunity of the upper respiratory tract, few mycobacteria might reach the alveoli, *Mtb* infection niche. Once the host immune system senses the presence of *Mtb*, it will initially mount an early innate immune response, followed by a delayed adaptive one, which can result in either *Mtb* eradication or survival.^106^ Generally, the onset of the host adaptive response results in the containment of the infection. Hence, the majority of the infected individuals will be asymptomatic and develop latent TB infection (LTBI),^107^ while in a few infected individuals, there will be progression toward an active form of the disease. Overall, pulmonary TB is the most common form of infection. However, up to 20% of infected individuals will develop extrapulmonary or disseminating TB.^108^ In particular, disseminating TB may occur when *Mtb* accesses the systemic circulation and starts to multiply, thus affecting several organs, such as the meninges, finally resulting in the development of CNS‐TB.^108^

CNS‐TB is a clinical condition that occurs once *Mtb* reaches the meninges, where this successful pathogen starts to duplicate, causing local inflammation. CNS‐TB is the second leading most common cause of meningitis, and it is associated with high mortality when diagnosed and treated, and can be fatal if left untreated. It is estimated that 0.3%–4.9% of all people diagnosed with TB have CNS‐TB, hence suggesting that 30,000–490,000 people develop CNS‐TB each year. Furthermore, factors such as the prevalence of pulmonary TB, age, and HIV infection can all possibly determine the geographical incidence and variability of CNS‐TB.^109^

Because of the absence of proper diagnostic tools, the incidence and mortality of CNS‐TB are heavily under‐reported. In this regard, a meta‐analysis by the World Health Organization (WHO)‐TB notifications modeled that, in 2019, CNS‐TB affected 164,000 adult individuals, among which 23% was showing HIV/TB co‐infection, causing 78,200 deaths (48% of incident CNS‐TB).^110^ Additionally, a reduction in Bacillus Calmette‐Guérin vaccination in newborns has been associated with an increase in childhood CNS‐TB, even in high‐resource countries.^111^

It is noteworthy that despite the current advancements in diagnostic tools to confirm TB infection, confirming CNS‐TB remains challenging. A fast and inexpensive test to confirm CNS‐TB is the Ziehl–Neelsen staining (i.e., an acid‐fast staining technique commonly used to stain mycobacterial species) in the cerebrospinal fluid (CSF).^112^ However, a clinical study performed on 618 ethnically diverse CNS‐TB‐positive patients showed that CSF Ziehl–Neelsen staining appears to have a low sensitivity (approximately 30%).^113^ Currently, to diagnose CNS‐TB, WHO recommends the GeneXpert MTB/RIF Ultra test, which is rapid and allows, at the same time, the identification of rifampicin resistance. Although GeneXpert MTB/RIF Ultra is used to diagnose extrapulmonary TB and its utility in assessing CNS‐TB, this test is scarcely available in low‐ and middle‐income countries.^112^ Next to the GeneXpert MTB/RIF Ultra additional other tests for CNS‐TB are currently available, such as the loop‐mediated isothermal amplification, the test‐tube‐based DNA amplification technique, and the semiautomated chip‐based PCR assay Truenat MTB.^112^

Clinically, CNS‐TB shows a wide spectrum of symptoms such as altered mental status, meningitic features, seizures, cranial nerve palsies, and focal neurological deficits. Overall, *Mtb* invasion of the CNS triggers an aberrant host‐immune response that results in inflammation and damage of brain tissue and the meninges. Indeed, aside from the bacilli directly challenging the microglia, neurons, and astrocytes through antigen recognition, the clinical spectrum of CNS‐TB depends on the host immune response.^111^, ^112^ Although the mechanism(s) driving CNS‐TB physiopathology are not yet fully elucidated, CNS destruction has been linked to a disequilibrium of pro‐inflammatory and anti‐inflammatory cytokines, which depends on the bacillary load.^110^, ^113^ In more detail, a high CSF bacillary load has been associated with severe CNS‐TB and a two‐fold increase in mortality, while patients with low CSF bacillary load have a lower risk of death and may benefit from additional supportive care.^110^, ^114^, ^115^

Currently, the WHO guideline recommends antitubercular chemotherapy for the treatment of drug‐susceptible CNS‐TB infections, albeit with an extended duration. Treatment of CNS‐TB patients includes a 2‐month four‐drug cocktail (i.e., rifampicin, isoniazid, pyrazinamide, and ethambutol), which is then continued for an additional 10 months with only isoniazid and rifampicin.^110^, ^116^ Indeed, CNS‐TB treatment and *Mtb* clearance are further complicated by the reduced permeability of the BBB to several first‐ and second‐line antitubercular drugs.^117^

One of the hallmarks of TB is the formation of the granuloma—a cluster of diverse immune cells formed in response to chronic infectious or noninfectious stimuli. Although the role of the granuloma in either protecting or promoting the dissemination of *Mtb* remains an open question, it is well known that *Mtb* adapts to the harsh host microenvironment by entering a quiescent dormant state.^118^ To date, the precise niche(s) where dormant mycobacteria hide remains elusive. Interestingly, considering the high CNS inaccessibility and its lower immune surveillance, CNS might indeed represent a unique advantageous microenvironment where *Mtb* can escape immune recognition and thus hide.^117^ In 1933, Rich and colleagues identified within the brain parenchyma and meninges of *Mtb*‐infected individuals the presence of granulomas, later named Rich foci,^119^ which start to appear during the initial hematogenous dissemination of the bacilli seeded in the brain parenchyma and meninges.^119^ Several observations have been made regarding the Rich foci formation, rupture, and development and progression of CNS‐TB. Yet, the mechanism(s) used by *Mtb* to enter the CNS remain unclear. In particular, how *Mtb* can cross the BBB continues to be an open question.

**Figure 6:**
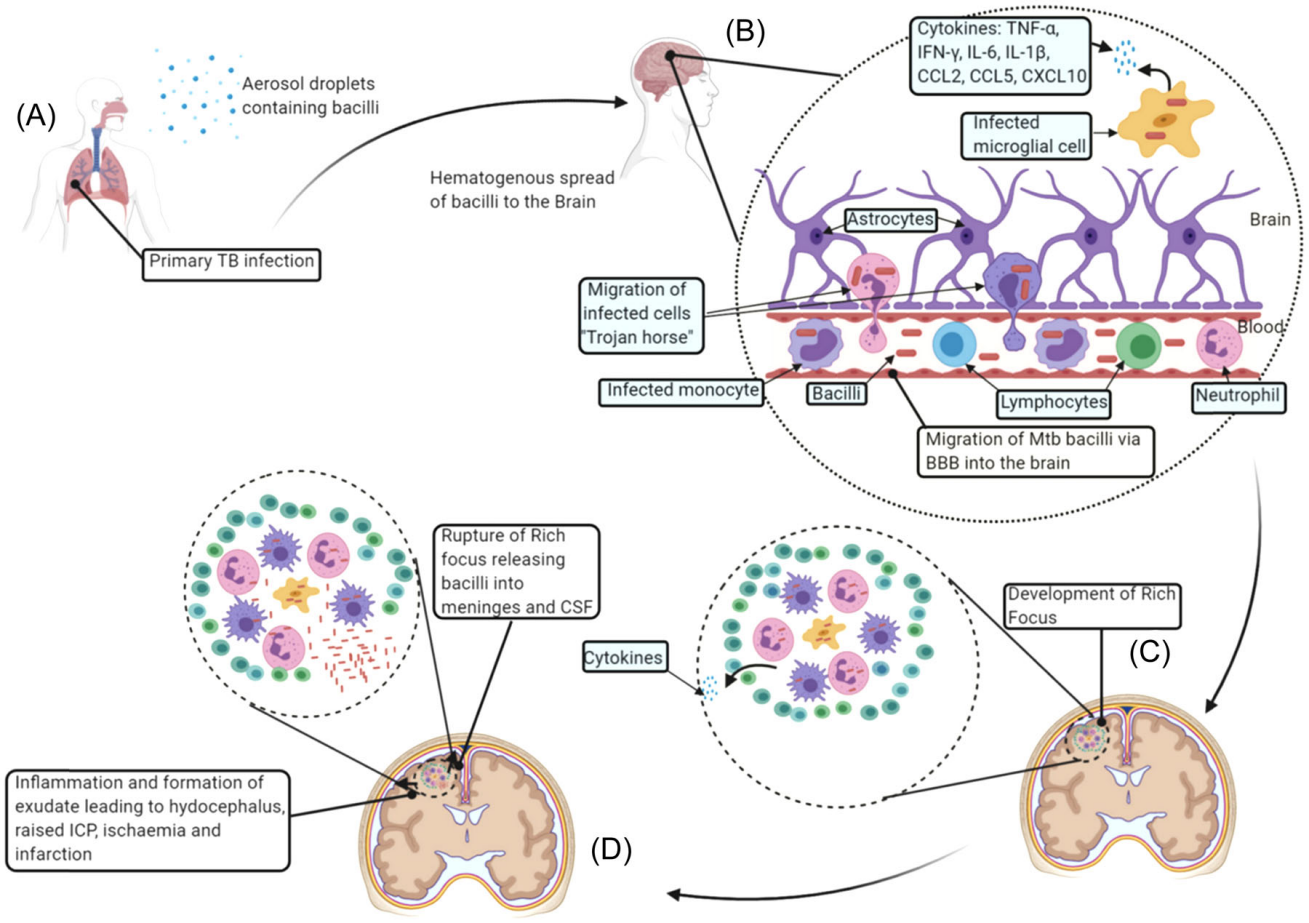
The generalized pathogenesis of tuberculous meningitis. (A) The host inhales aerosol droplets containing *M. tuberculosis* (Mtb) bacilli. Within the lungs, the bacilli may infect the alveolar macrophages, resulting in the formation of granuloma. The bacilli may then escape from a damaged granuloma or the lungs during primary TB, causing bacteremia, resulting in a hematogeneous spread of the bacteria into the brain. (B) Extracellular bacteria and infected cells may pass through the blood–brain barrier (BBB) into the brain. Once in the brain, the bacilli infect microglial cells, which then, together with infiltrating cells, release cytokines and chemokines, leading to disruption of the BBB and influx of other uninfected immune cells into the brain. (C) This results in the formation of the granuloma “Rich focus.” (D) When the Rich focus ruptures, the bacteria are released into the subarachnoid space, leading to the dissemination of the infection to the CSF and meninges. The release of bacteria into the meninges and CSF leads to meningeal inflammation and the formation of thick exudate. The thick exudate precipitates TBM signs. This could be the image to use for the CNS‐TB part. Image and caption courtesy of C. M. Manyelo et al., “Tuberculous Meningitis: Pathogenesis, Immune Responses, Diagnostic Challenges, and the Potential of Biomarker‐Based Approaches”, Journal of Clinical Microbiology 59 (2021). This is an open‐access article distributed under the terms of the Creative Commons Attribution 4.0 International license. [Color figure can be viewed at wileyonlinelibrary.com]

By definition, the BBB is a highly selective permeable layer that protects the CNS from systematic circulation. It is composed of a layer of specialized endothelial cells held by tight junctions, which are surrounded by and closely interact with astrocytes, pericytes, and microglia. Because of the BBB physical properties, this barrier selectively reduces the penetration of circulating substances and/or infectious agents to the CNS. Nevertheless, several pathogens, such as *Mtb*, have all the necessary machinery to adhere to such cells and cross the BBB, finally causing neuroinfections.^118^, ^119^ To cause CNS granuloma formation and consequently CNS‐TB, *Mtb* must cross the BBB. Thus far, three possible diverse BBB crossing strategies have been proposed: (i) transcellular migration, (ii) paracellular migration (i.e., disruption of the BBB mediated by the secretion of bacterial toxins), and (iii) the Trojan horse mechanism (Figure 6). Of these, here, we will be focusing on the transcellular migration and Trojan horse mechanisms.

#### 2.4.1 Transcellular migration

Pathogens‐ BBB transcellular migration is a receptor‐mediated process previously described for *Streptococcus pneumoniae, Haemophilus influenzae*, and *Neisseria meningitidis* and is mediated by endothelial cell endocytosis.^118^, ^120^ Endothelial cells are not typically phagocytic cells. However, in the presence of microbes, they can elicit an antibacterial response, producing and releasing antimicrobial peptides.^121^ In this regard, diverse experimental observations have been pointing out the key role played by brain endothelial cells in promoting *Mtb* BBB crossing independent of infected leukocytes and/or macrophages.^122^ In particular, the role played by BBB‐associated endothelial cells has been investigated in *Mycobacterium avium* i.v.‐infected mice. Here, it has been shown that the bacteria might cross the BBB by invading the epithelial cells in the choroidal plexus and not by crossing the tight junction holding those cells together.^122^ Moreover, a second in vitro study performed on *Mtb*‐infected human brain microvascular endothelial cells (HBMECs) reported that the mycobacterial invasion and movement required HBMECs to rearrange their actin cytoskeleton.^123^ Overall, all the evidence collected so far supports the hypothesis of *Mtb* encoding putative virulence factors that might promote CNS invasion. Although such drivers remain partly uncharacterized, pknD (Rv0931c)—a “eukaryotic‐like” serine–threonine protein kinase—was identified as a key mycobacterial protein promoting *Mtb* CNS‐TB progression.^117^ Interestingly, those in vitro data demonstrate that the sensor domain of PknD might promote mycobacterial adhesion with specific brain laminin isoforms (i.e., laminin 1 and 2), thus^120^ further confirming free *Mtb*'s ability to cross the BBB, by possibly invading the endothelial cells and damaging the basal lamina. A second study aiming to identify the molecular mechanism(s) dictating *Mtb* BBB transcellular migration focuses on understanding the role of ESX‐1 in *Mycobacterium marinum* in regulating CNS macrophage‐independent crossing. To probe the role of ESX‐1 secretion in CNS invasion, van Leeuwen and colleagues used an *M. marinum esx‐1* mutant, and using correlative light‐electron microscopy (CLEM), they showed that the *esx‐1* mutant was mostly located in the lumen of the blood vessel surrounding the Zebrafish CNS, and, contrary to the wild‐type strains, this mutant was unable to cross or damage the basal lamina. Hence, this proves that the ESX‐1 secretion system plays a crucial role in regulating the mycobacterial CNS transcellular migration.^124^

#### 2.4.2 Trojan horse

The Trojan horse mechanism accounts for *Mtb*'s intracellular pathogenic nature, which results in the mycobacterial ability to survive and replicate within the macrophages.^123^, ^124^ Macrophages ability to restrict mycobacterial growth only occurs in the presence of lymphocytes and/or cytokines (such as interferon‐γ), suggesting a key role of adaptive immunity in restricting bacterial dissemination and survival. This indicates that mycobacteria can exploit host macrophages as a specialized niche to survive.^125^ When *Mtb* reaches the alveolar microenvironment, the bacteria are quickly ingested by the alveolar macrophages, which are then attacked by the pathogen to get transported across the alveolar wall and reach the bloodstream. Indeed, an in vitro study performed by coculturing A549 human type II alveolar epithelial cells with human *Mtb*‐infected monocytes demonstrated that not only were mycobacteria able to infect the epithelial cells but also that this induced an increase in infected monocytes translocation, which overall depended on an increase in MCP‐1 chemokines.^126^ In this scenario, in the early stage of the infection, *Mtb* might promote macrophage recruitment to the infection site, hence transporting the infected macrophages back into the tissues and allowing the pathogen to gain access to deeper tissue. This model further provides a scenario where mycobacteria might use macrophages as an initial niche to reside in, to then induce their lysis and extracellular dissemination.^125^ Nevertheless, mycobacterial dissemination through host macrophage migration could also be a mechanism used by the host to promote the priming of dendritic cells, finally leading to the onset of the adaptive immune response.^127^

### 2.5 NPs role in the treatment of brain infection

Meningitis—defined as infection and inflammation of the fluid and membranes surrounding the brain and the spinal cord—is commonly caused by diverse infection agents, such as viruses (e.g., enteroviruses HIV, herpes simplex viruses [HSV], and mumps virus) and bacteria (e.g., *Streptococcus pneumoniae, Neisseria meningitidis, Haemophilus influenzae*, and *Mycobacterium tuberculosis*).^128^ Although infectious‐mediated meningitis is treated by a combination of antimicrobials, to eradicate the pathogen, and corticosteroids, to modulate the inflammatory response, this chemotherapy still has several drawbacks. Among these is the limited BBB permeability of multiple antimicrobials used to treat bacterial infection.^129^ The advent of nanomedicine‐based therapies allowed to overcome this limitation. Indeed, the use of nano‐dimensional NPs enabled curing of brain inflammation disorders, by enhancing drug(s) administration across the BBB.^130^ Because of NPs features (i.e., elevated surface‐to‐volume ratio, easy surface functionalization, and ability to cross biological barriers, such as the BBB), NPs have been proposed as powerful novel therapeutic tools to treat and enable the early diagnosis of meningitis. Some of the main NP systems that are currently being used will be briefly described here.

#### 2.5.1 Lipid‐based NPs (I.e., liposomes)

Because lipid‐soluble molecules can easily penetrate the BBB through passive diffusion, liposomes represent an optimal NP system to carry both hydrophilic and hydrophobic antimicrobial(s) into the brain and hence induce the clearance of the infection.^131^

#### 2.5.2 Metal NPs

To date, gold (AuNPs) and silver (AgNPs) NPs are the most used NPs in drug delivery. Because of AuNPs tunable physical and chemical properties, they are widely investigated as a possible new photodynamic treatment for brain diseases, such as, for instance, *Naegleria fowleri* infection.^130^, ^131^ Similarly, because of their spherical nanometric dimension and biocompatibility, AgNPs have been considered excellent drug delivery carriers, which can diffuse through the BBB, permitting the accumulation of the desired drug(s) at the site of interest.^87^ AuNPs and AgNPs ability to cross the BBB has been recently observed for the treatment of brain infections. Both metallic NPs were loaded with diverse antimicrobials and, once administered, showed antibacterial activity against diverse possibly neuropathogenic bacteria, such as *Escherichia coli* K1 and *Staphylococcus aureus* (MRSA).^132^

#### 2.5.3 Polymeric NPs

Polymeric NPs are considered highly compatible with the body cells and thus easily biodegradable. Because of those properties, polymeric NPs have been widely used to generate diverse drug delivery systems to treat several bacterial pathogens. Indeed, diverse experimental observations proved that polymeric NPs increase BBB drug(s) permeation and promote the eradication of bacterial‐induced meningitis.^132^, ^133^ Regarding CNS‐TB treatment, because antitubercular drugs show limited ability to cross the BBB, diverse polymeric NPs have been generated to improve their delivery to the CNS and resolve the infection.^134^ Among them, it is worth mentioning a PLG NP system in which three to four anti‐mycobacterial drugs (i.e., isoniazid, rifampicin, pyrazinamide, and ethambutol) can be encapsulated. Interestingly, this NP formulation can be administered orally, with a single dose sustaining an effective drug concentration in the brain for 9 days. Overall, Pandey et al.^135^ demonstrated that five administrations performed every 10 days resulted in the complete eradication of *Mtb* H37Rv in mice meninges.

#### 2.5.4 Extracellular membrane vesicles (EMVs) and nanomicelles

EMVs can be defined as lipidic bi‐layered structures organized in a spherical manner, which can be used to deliver a specific cargo (i.e., drug) within the same microorganism or mammalian cell. Specifically for the treatment of brain bacterial‐induced infection, trivalent native outer membrane vesicles were genetically engineered from *N. meningitis* serogroup B and used to induce an immune response first, followed by immunization, in an infant rhesus macaque model.^136^

Self‐assembly nanosystems of either amphiphilic surfactant or amphiphilic copolymers that can incorporate drug(s), namely, nanomicelles, have been used recently to induce the accumulation of BBB inefficiently transported drugs within the brain.^129^ In this regard, several efforts have been made to generate engineered nanomicelles to enhance brain targeting (i.e., RVG_29_ and p‐glycoprotein inhibitor (Pluronic® P85 unimers) superficial functionalization) and induce pneumococcal meningitis clearance.^137^

## 3 CONCLUDING REMARKS

The topics discussed in this chapter are probably the “tip of the iceberg”. It is evident that NPs have the clear advantage (and yet the most challenging aspect) of an interdisciplinary area of research, which can simultaneously be dangerous: if not properly balanced, it can be rather superficial, even overlooking critical details in favor of the final applications. Our goal is to translate the acquired knowledge to the clinic as soon as possible, without jumping from fundamental to applied science too quickly and without analytical control.

An additional aspect to be considered is that any new molecule will have to be subjected to intensive clinical testing with considerable time and economic investment, typically between 5 and 20 years, and between a few and several thousands of millions of pounds/dollars/euros. Inevitably, together with the increase in the production of new medicines and devices, the large clinical data make future decisions even more stringent. This is quite evident in the pharmaceutical industry, where the number of new drugs has been increasing steadily, but the cost associated with new drugs has increased exponentially. In 1998, the average R&D cost for a new molecular entity was estimated at around $26 billion (inflation corrected), while in 2008, it was already estimated to be c.a. $50 billion.

NPs safe development for biomedical applications is still far from these numbers. Medicinal chemistry, nowadays, is not only a synthetic chemistry affair: drug design is continuously aided by bioinformatics, genomics, proteomics, cell‐based screens, and HTP approaches. Drugs are synthesized in libraries, and these are tested in output protocols that enable fast discovery and mapping of fundamental structure‐to‐function relations. Although there are few examples of HTP approaches providing new insights into biomaterial design, these do not still match with more mechanistic studies to create fundamental principles for future biomaterial design.

When a specific material is selected for creating NPs for biomedical applications, their final use, the safety of the raw ingredients, and the processing of production must all be a priori designed.

## AUTHOR CONTRIBUTIONS

All authors contributed to the study's concept, design, and work, as well as the data analysis and interpretation. All authors edited, reviewed, and approved the final draft of the manuscript.

## CONFLICT OF INTEREST STATEMENT

Prof. Loris Rizzello is the associate editor of Ibrian; he is not involved in peer review. The remaining authors declare no conflict of interest.

## ETHICS STATEMENT

Not applicable.

## Data Availability

The data of our study are available on reasonable request.
